# What You Should Remember in Managing Pilonidal Disease

**DOI:** 10.3389/fsurg.2021.792121

**Published:** 2021-12-07

**Authors:** Diego Segre

**Affiliations:** Formerly Responsible Colonproctology Unit, S.Croce Hospital, Private Practice, Cuneo, Italy

**Keywords:** pilonidal sinus disease, patients information, patients decision-making, consensus protocol, pilonidal sinus wounds

## Introduction

Most articles on pilonidal sinus disease (PSD) published in the last two decades have reported an incidence ranging from 20 to 30 in 100,000 people, with males affected twice as often as females (1, 2). In recent reports, concerning German and Turkish population, global incidence rates appear to be increasing in almost all age groups and both sexes with the burden of this disease exceeding those of inguinal hernias (3, 4). Still today there is no clear consensus on what constitutes optimal treatment resulting in rapid healing and minimal complications. Actually PSD has a wide spectrum of presentations ranging from minimally symptomatic pits at the natal cleft to massive abscess formation with or without complex tracts and fistulas. The importance of individualizing surgical management, tailoring it to the specific case of PSD, has been established (5). Most research has addressed the technical aspects of surgery and treatment but what seems to be lacking are clear recommendations both about pre-operative information to patients and shared decision-making and about patients' and families' involvement in post-operative care and other problems that may arise. These aspects have been constantly overlooked in current guidelines and consensus statements from scientific societies (2, 5, 6).

## Practical Aspects of Management

### Information

Patients are often reluctant to address this condition and generally do not have a clear understanding of what to expect following excision of their pilonidal sinus. Embarrassment often inhibits communication with the surgeons and is responsible for inadequate knowledge about surgery and recovery time.

The problem is that patients are not always involved in the choice of surgical treatment. They are often unconcerned about the potential burden of post-procedural wound care, the support they will need from others or the risk of post-procedural pain and recurrence.

Patients should receive detailed verbal and written information, tailored to their needs, at the right time.

The explanation should not only deal with surgical techniques, it should manage patients' expectations about aftercare and the uncertainties which surround clinical outcomes. Also advantages and disadvantages of different approaches should be communicated as well as time requirements, tasks and psychosocial issues associated with recovery.

Individuals with simple asymptomatic sinuses may be asked to wait for their condition to deteriorate going on with conservative therapy (7). In fact, PSD is a self-limiting condition that disappears with age (usually by 30 years). Conservative non excisional therapy (meticulous hair control by natal cleft shaving, weight loss, improved perianal hygiene and limited lateral incision and drainage for abscess) has demonstrated effective control of PSD while promoting near-normal work status (8). Equally, Isik et al. (9) have reported excellent results in selected cases with the use of fibrin glue without surgery. As regards children, who abitually spend long periods at home at the time when PSD emerges, sitting at the computer and/or playing computer games for many hours a day, they should be advised to spend less time sitting. Conservative approach does not require days off school: patients do not need to stay in hospital or to come to clinic every day, so their life quality does not get worse. It should be considered the first choice initial treatment for adolescents with PSD, with the advantage to be cost-effective (10). Patients operated for the first time are often overly optimistic about the chances of success, while those with recurrent disease sometimes regret poorly informed decision and exhibit higher psychosocial burden (7).

### Shared Decision-Making

Both shared decision-making and the consent process itself are compromised if patients are poorly informed about their condition.

Treatment decisions may sometimes be challenging when there are many available treatments supported by variable evidence and the probability of various outcomes. Actually a substantial and complex information can sometimes be difficult to understand and may lead to distress (7).

Even if patients feel involved in decision-making, their expectations may not be met if they are not fully informed about a care pathway (5): information gaps can considerably reduce patient's ability to self-manage (7). Surgeon experience and confidence in performing the different techniques should also be part of the decision equation (2). When a flap-based procedure is programmed, an explanation of the potential cosmetic outcome resulting from surgery should not be left out and difference in cosmetic alterations between surgical approaches should be explained (2). Limberg flap technique, for example, can leave an unsightly scar across the midline with natal cleft distortion resulting in an asymmetry of the gluteal contour. Few data exist about post-operative perception of body image and cosmesis scores after PSD surgery. According to Sengul et al. (11), however, patients who have suffered PSD are mostly far more concerned about the reconstruction of their functional problems due to their chronic disease than the cosmetic outcome.

### Post-operative Care

Proper post-operative care is very important to improve, as much as possible, individuals' experiences with a pilonidal sinus wound and its impact on all activities of living (12). It also plays an essential role in the prevention of recurrence, which can occur up to 20 years following initial surgery (13). The problem in general does not arise in patients submitted to minimally invasive approaches both traditional (Lord-Millar, Bascom, Gip's etc.) (14) and endoscopic (EPSiT, VAAPS etc.) (15, 16) and after closed techniques (2). Once given adequate instructions, better if written, parents or motivated friends will manage without difficulties or risks simple daily wound dressing using ordinary disinfectants. Hair growth may be easily controlled by shaving, depilatory creams, waxing or regular laser in and around natal cleft; it has to be continued every 7–10 days until healing occurs and beyond, for several months (6).

Both direct midline and off-midline closure are associated with a rather high rate of complications and wound dehiscence [up to 45% after Limberg flap (17)].

Following failed primary closure and when sizeable open wounds are left for healing by secondary intention, care may be particularly demanding. Wound healing can take over 6 months and all activities are affected, from personal cleansing and dressing to mobilizing, expressing sexuality, working and playing. For these reasons it should not be managed empirically. Although healing times seem not to be affected by the dressing types used (11), care should ideally follow an evidence-based protocol with guidelines covering all aspects of post-operative care. It should therefore include precise instructions about optimal positioning for wound assessment and care, use of systemic and topical antibiotics, local wound interventions, wound cleansing, peri-wound skin care, pain control, nutrition and pre and post-healing physical activities (18–20).

## Conclusions

Research on PSD has been generally technical and procedural, mostly pertaining to surgical management (12). What seems to have been overlooked is the conscience of the impact this disease may cause on life activities in patients who are usually young, healthy, in their most productive years. Actually patients facing this condition usually tend to underestimate many aspects of it such as the time needed for post-surgical wound healing, the burden of wound care and the risk of recurrence (7). Hence the importance to make sure that they might receive comprehensive verbal and written information both about available surgical approaches and about proper post-operative wound care they will need. Research has not yet standardized an optimal management for complex wounds after PSD surgery (7), further studies are required to formalize an evidence-based protocol patients can follow.

## Author Contributions

The author confirms being the sole contributor of this work and has approved it for publication.

## Conflict of Interest

The author declares that the research was conducted in the absence of any commercial or financial relationships that could be construed as a potential conflict of interest.

## Publisher's Note

All claims expressed in this article are solely those of the authors and do not necessarily represent those of their affiliated organizations, or those of the publisher, the editors and the reviewers. Any product that may be evaluated in this article, or claim that may be made by its manufacturer, is not guaranteed or endorsed by the publisher.
